# Nurse Managers’ Experiences and Competency Requirements in the Care of Patients with Emerging Infectious Diseases: A Meta-Synthesis

**DOI:** 10.3390/healthcare14142088

**Published:** 2026-07-13

**Authors:** Zhengji He, Qun Xiao, Minye Li, Chen Zhi, Hui Ma

**Affiliations:** 1 School of Nursing, Southern Medical University, Guangzhou 510515, China; h20100318@smu.edu.cn (Z.H.); xq22420255@smu.edu.cn (Q.X.); 2 Department of Nursing, Chinese People’s Liberation Army General Hospital, Beijing 100853, China; myli233@163.com

**Keywords:** nurse managers, emerging infectious diseases, core competencies, support systems, meta-synthesis

## Abstract

**Objective:** This study seeks to synthesize the experiences, challenges, and core competency requirements of nurse managers caring for patients with emerging infectious diseases to inform targeted training and strengthen emergency response systems. **Background:** The frequent emergence of emerging infectious diseases has placed unprecedented strain on global public health systems, with nurse managers playing a pivotal role in coordinating and sustaining outbreak responses. **Methods:** A literature search was conducted across PubMed, CINAHL (EBSCO), Embase, Web of Science, Scopus, CNKI, and other databases. Qualitative studies were screened and assessed using the JBI appraisal tool. Thirteen studies were included and meta-synthesized. Thematic synthesis was used to synthesize the findings. **Results:** Four main themes emerged: multidimensional experiences of nurse managers, dual pressures from resource shortages and environmental constraints, core competency requirements, and the need for organizational and system support. **Discussion:** Outbreaks intensified physical and mental strain, increased burnout and turnover, yet also fostered professional growth. Systemic support, flexible staffing, digital training, and inclusive governance are needed to ensure well-being, care resilience, and leadership retention. **Conclusion:** This study outlines challenges and competencies of nurse managers during health crises, underscoring the need for comprehensive support to guide specialized training and improve public health preparedness.

## 1. Introduction

Emerging infectious diseases (EIDs) refer to diseases that are newly discovered, have been declared eradicated but re-emerge, or suddenly appear with the potential to cause significant public health hazards and threats. These diseases are characterized by their sudden onset, rapid transmission, high infectivity, and a tendency to spread rapidly and widely [1]. They present challenges including insufficient understanding of transmission patterns, unclear clinical outcomes, and a lack of established treatment protocols. Taking the novel coronavirus infection (Corona Virus Disease 2019, COVID-19) as an example, as of February 2025, there were 770 million confirmed cases worldwide, with a death toll of 7.08 million, posing a severe threat to human life safety and social stability [2].

As the primary caregivers in EID treatment, healthcare workers are often the first point of contact with patients, placing them in a critically important position. They can significantly contribute to infection risk assessment, transmission control, and infection rate reduction [3]. Nurse managers, as core members of the medical team, are responsible for coordinating the work environment, ensuring the safety and well-being of nursing staff, and organizing task allocation [4]. Their effectiveness in these roles is not solely a matter of individual skill, but rather, it is fundamentally shaped by their structural position within the healthcare organization.

According to Kanter’s theory of structural empowerment, an individual’s ability to perform effectively and demonstrate leadership is determined by their access to information, support, resources, and opportunities within the workplace [5]. During EID outbreaks, nurse managers often face systemic constraints in these very areas, such as fragmented information flows, limited decision-making autonomy, resource scarcity, and inadequate organizational support, which can severely hinder their capacity to lead and sustain team resilience. Amid pandemic pressures, nurse managers are required to balance dual obligations, namely the assurance of patient safety and the maintenance of care quality, as well as the safeguarding of clinicians’ welfare. This is further compounded by the profound emotional burdens experienced by healthcare professionals, including fear, uncertainty, and the helplessness of failing to provide compassionate care for patients with EIDs [6]. Their professional competencies and response strategies directly influence the effectiveness of care and patient outcomes. Additionally, their experiences involve a complex interplay of managerial, leadership, emotional and professional factors, meaning that their needs are unique. Drawing on Kanter’s theory, we believe that the lack of systemic support may be a key but underexplored factor shaping the experiences of nurse managers. This meta-synthesis therefore aims to identify not only the challenges faced by individuals, but also the structural gaps that urgently need to be addressed.

A significant body of research has documented the clinical experiences of frontline nurses during EID outbreaks, particularly in the initial phase [7,8]. However, limited attention has been given to the unique pressures and training needs of nurse managers [9]. It remains unclear what specific challenges nurse managers face during EID outbreaks, how their competency requirements differ from those of frontline nurses, and what organisational support they require to maintain their leadership and well-being. Even within the same context, the experiences of nurses and nurse managers may differ due to their distinct roles [10]. A mere descriptive summary of existing findings is insufficient. Therefore, a thematic synthesis is required to identify cross-cutting patterns and generate actionable insights. To address this gap, this meta-synthesis is guided by the following research question, formulated using the PICoS framework: What are the experiences and core competency requirements of nurse managers caring for patients with EIDs in acute healthcare settings?

The objective of this study is to methodically synthesize and analyze the extant qualitative evidence concerning the experiences and competency requirements of nurse managers in the care of patients with EIDs. By integrating findings from a variety of contexts, it seeks to develop a comprehensive interpretive understanding of the challenges they face and the competencies deemed essential. The synthesis of evidence is intended to provide a foundational theoretical basis for informing future research, policy development, and the design of targeted support systems and training programs.

## 2. Data and Methods

### 2.1. Protocol Registration and Reporting Guidelines

This systematic review protocol was registered with PROSPERO (registering number: CRD420251073047). This study was conducted and reported in accordance with both the Preferred Reporting Items for Systematic Reviews and Meta-Analyses (PRISMA) checklist and the Enhancing Transparency in Reporting the Synthesis of Qualitative Research (ENTREQ) statement to ensure methodological transparency and rigor.

### 2.2. Search Strategy

The JBI three-step search strategy was used for literature retrieval. In the first step, we conducted an initial literature search of PubMed, CINAHL (EBSCO) and CNKI Database to identify titles, abstracts, and keywords to generate a search strategy. In the second step, we combined the theme titles with free-text keywords before conducting a thorough computerized search of Chinese and English databases, including PubMed, CINAHL (EBSCO), Embase, Web of Science, Scopus, ProQuest, SinoMed, CNKI Database, Wanfang Data and VIP Database. In the third step, we identified all references from the included literature to supplement other relevant literature. We also manually screened the reference lists of all included studies and relevant review articles to identify additional studies not captured by the electronic database searches. The search period was from the establishment of the database to March 2025. Comprehensive search strategies for each database, including the specific query syntax and keywords, are provided in Supplementary Material File S1.

### 2.3. Literature Inclusion and Exclusion Criteria

The inclusion and exclusion criteria for the studies were established based on the PICoS framework recommended by the Australian JBI Centre for Evidence-Based Healthcare. The inclusion criteria were as follows: (a) Population: Nurse managers involved in the care of patients with EIDs; (b) Phenomenon of interest: Experiences, perceptions, and training needs of nurse managers regarding the care of patients with EIDs; (c) Context: Healthcare settings during outbreaks of EIDs; (d) Study design: Qualitative methods such as phenomenology, grounded theory, ethnography, or qualitative descriptive research. The exclusion criteria were as follows: (a) Studies published in languages other than English or Chinese, as the research team is proficient only in these two languages, to ensure accurate data extraction and interpretation; preliminary searches indicated that most eligible studies were published in these languages; (b) Literature with incomplete full-text access or missing data; (c) Duplicated publications; (d) Studies not focused on nurse managers’ experiences or competency requirements, including those focusing solely on frontline nurses without addressing nurse managers‘ perspectives, or those focusing on clinical treatment outcomes rather than management experiences, as these do not address the research question.

### 2.4. Literature Screening and Data Extraction

All retrieved records were imported into EndNote 20 software for bibliographic management. Two researchers (HZJ and XQ) with expertise in evidence-based medicine independently conducted literature screening and data extraction, with cross-checking. In the event of disagreement, a third researcher (LMY) was consulted for judgment.

The screening process consisted of two stages. In the first stage, HZJ and XQ independently screened all titles and abstracts, excluding clearly irrelevant records to the research topic. In the second stage, the same two reviewers independently assessed the full texts of the remaining articles to determine eligibility based on inclusion criteria. Disagreements at either stage were resolved through discussion. If consensus could not be reached, a third reviewer was consulted for a final decision.

We used the JBI Qualitative Assessment and Review Instrument (QARI), a data extraction tool for qualitative research, to extract the findings. A self-designed table was used to extract the general information from the included studies. The data extracted included geographical location, number of participants, hospital setting, research design and method of data collection. Data extraction was performed independently by HZJ and XQ, with cross-checking to ensure accuracy.

### 2.5. Literature Quality Assessment

Using the JBI critical appraisal tool [11], each study was appraised for methodological quality by two independent reviewers (HZJ and XQ) and checked by a third reviewer (LMY). Any disagreements between reviewers were resolved through discussion or by consulting the third reviewer. The checklist consists of 10 items, each rated as “Yes,” “No,” “Unclear,” or “Not Applicable.” We calculated the total score by awarding 1 point for each ‘yes’ and 0 points for ‘no’ or ‘unsure’. The studies were then graded according to their total score: 9–10 points was Grade A, 5–8 points was Grade B, and 0–4 points was Grade C. We treated all items equally and did not designate any subset as key items in advance. Ultimately, only those studies graded as Grade A or B were included [12].

### 2.6. Data Analysis Method

Thomas and Harden’s [13] three-stage thematic synthesis approach was used to synthesize data from the included studies.

Firstly, the original research findings were extracted and preliminary codes generated. The researchers systematically reviewed the full texts of the included studies and repeatedly read the results sections, including participants’ verbatim statements and the authors’ summaries. Findings relating to participants’ views, experiences, behaviours or decisions were extracted sentence by sentence and assigned concise preliminary codes. For example, a head nurse stated: “Even though you tell me I’ll be in full PPE, people are still getting COVID in full PPE.” The researchers extracted the core meaning from this statement and coded it as “concern”. Similarly, other original statements were coded as “emotional exhaustion”, “PTSD”, “physical exhaustion”, etc. All coding was carried out independently by two researchers.

Secondly, the codes were clustered into descriptive themes. The two researchers compared the meanings and similarities of the initial codes, grouping those with similar meanings into preliminary categories. For example, although the codes “concern”, “emotional exhaustion”, “PTSD”, and “physical exhaustion” differed in their specific manifestations, they collectively pointed to the dual psychological and physical burden experienced by nurse managers during the pandemic. Therefore, they were merged into a descriptive theme: “Dual physical and mental burden”. During this process, any discrepancies were resolved through a re-examination of the original text and discussion. Each sub-theme was provided with a preliminary definition, and representative original quotations were retained.

**Figure 1:**
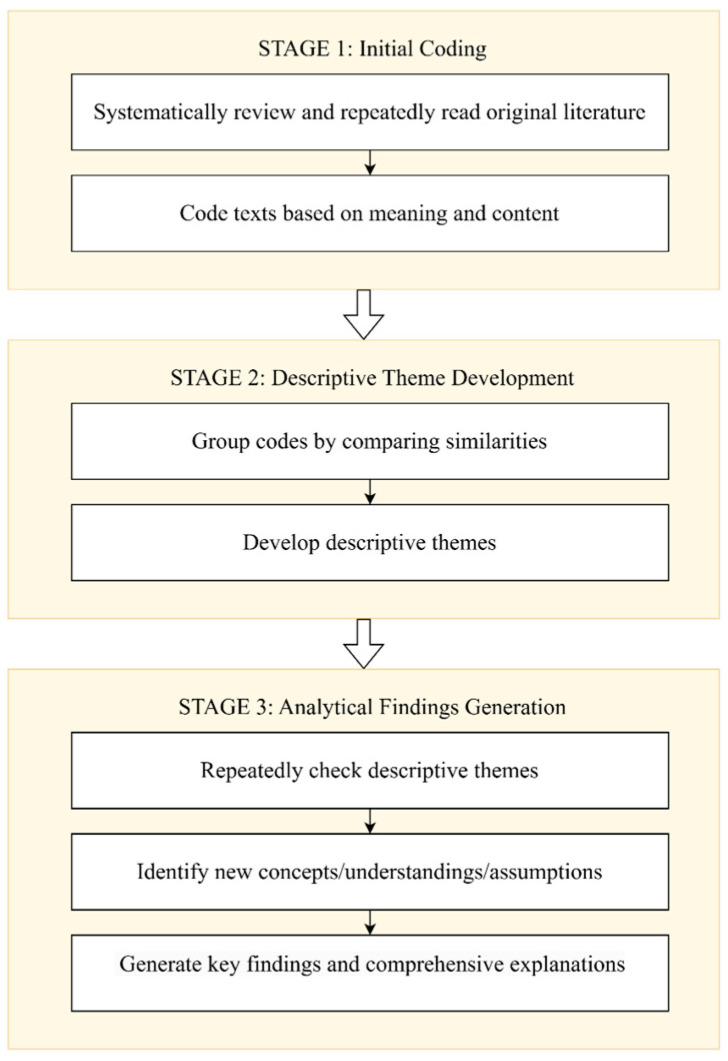
Flowchart of the process for synthesizing the data of the included studies.

Thirdly, descriptive themes were integrated to generate analytical themes. The researchers further examined the relationships between descriptive themes, moving beyond a direct restatement of the original findings to distil more interpretative analytical themes. For example, the descriptive theme “Dual physical and mental burden” was found to be intrinsically linked to other descriptive themes—together they reflected the multidimensional, compound challenges faced by nurse managers during the pandemic. Consequently, these descriptive themes were integrated into a single analytical theme: “Multidimensional experiences of nurse managers”. The specific process is shown in Figure 1.

## 3. Results

### 3.1. Search and Screening Results

**Figure 2:**
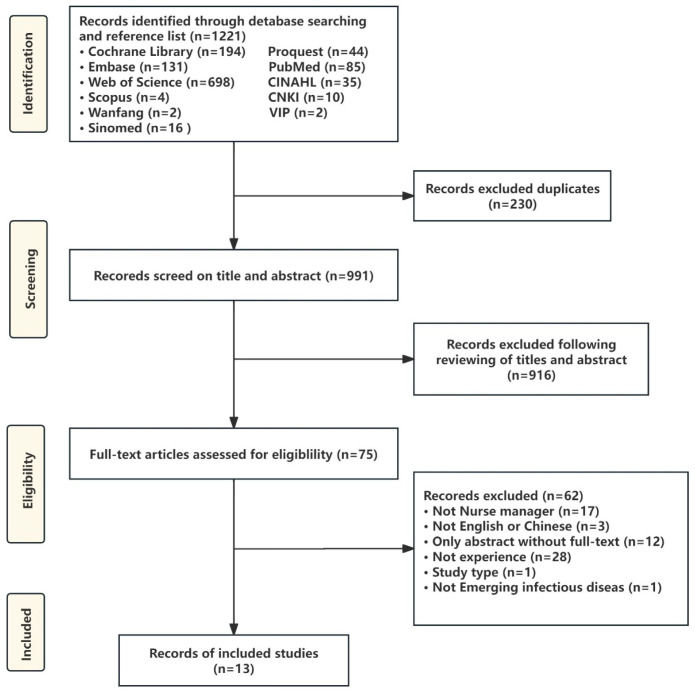
Flowchart of search results and study selection.

An initial search yielded 1221 potentially relevant records. After tracing references, removing duplicates, and screening full-text articles, 13 studies were ultimately included, including 2 in Chinese and 11 in English. The literature screening process and results are shown in Figure 2.

### 3.2. Basic Characteristics and Quality Evaluation Results of Included Literature

All of the included studies underwent quality appraisal using the JBI checklist, with each study rated as Level B. As shown in Table 1, no studies were excluded based on quality. In terms of methodological quality, the most common concerns across the included studies were related to Q1, where several studies received “Unclear” ratings, and Q6 and Q7, where most studies received “Unclear” or “No” ratings. On the contrary, all the studies performed well in Q2–Q5 and Q8–Q10, and almost all of them received the “yes” evaluation. This pattern indicates that although the included studies had methodological consistency in design and execution, the frequent lack of reflective statements is a significant limitation, which will be further discussed in the discussion section.

Among the thirteen included studies, the number of participants ranged from 8 to 37, with the majority being female, demonstrating the diversity in the scale of the studies (Table 2). Most studies employed qualitative descriptive and phenomenological methods, and the majority collected data through individual interviews.

### 3.3. Thematic Synthesis Results

A total of 13 studies were included, yielding 35 explicit results, which were categorized into 11 categories and integrated into 4 themes, as shown in Table 3.

**Theme 1: Multidimensional experiences of nurse managers**

This theme illuminates the complex personal and professional realities of nurse managers during health crises by integrating challenging and adaptive dimensions.

1. Dual physical and mental burden

Nurse managers frequently reported symptoms of somatization, including neck and shoulder pain, as well as sleep deprivation. Chinese nurse managers described persistent mental hyperactivity hindering rest [10], while Australian managers experienced “sleepless nights” due to shortages of protective gear [17].

In addition, nurse managers experienced widespread emotional exhaustion. They were concerned about the risk of infection for both themselves and their nurses (“Even though you tell me I’ll be in full PPE, people are still getting COVID in full PPE.” [17]). The high patient mortality rate left them heartbroken, and they grappled with feelings of helplessness, hopelessness, and exhaustion (“People were suffering, staff were suffering, and patients were dying at an alarming rate.” [16]). Some managers reported that they “might develop PTSD” [21], even having to confront the deaths of staff members [10,16,22].

They had to coerce nurses to exceed their work capacity, resulting in ethical dilemmas [17,19]. Nurse managers reported being compelled to deploy their staff to departments treating patients with EIDs or to accept involuntarily transferred staff, actions that conflicted with their leadership principles (“The staff were frightened, nervous, and crying.” [20]). Feelings of guilt arose from the work–family imbalance during the pandemic (“My daughter’s grades were down because she lacked enough supervision from me.” [10]).

2. Discrimination in society and the workplace

Ward managers experienced a sense of community among the staff who opposed them. South African managers reported being ostracized by colleagues, describing incidents of being ‘expelled from the meeting room,’ while nurses were stigmatized by the community as ‘virus spreaders.’ [19]. New managers felt compelled to accept an overwhelming workload (“but I couldn’t say no when assigned more work by a senior” [10]). Nurse managers indicated that they had also experienced stigma in their local communities (“Everyone was just too scared of nurses. They would just run away or avoid you. It felt like we were the ones who were carrying and spreading the virus.” [19]).

3. Positive attitude

Despite facing immense pressure, nurse managers continue to grow in adversity. They experience the power of collaboration, take pride in their professional roles and have a deep understanding of the importance of nursing work.

In the treatment of EIDs, nurse managers not only accumulated valuable experience in maintaining physical and mental well-being, but also transformed these practical insights into a foundation for optimizing future nursing management [14,15,17,20]. Multidisciplinary collaboration reached “unprecedented heights in nursing history” [24], and this team effort not only ensured high-quality patient care and safety but also built a mutual support network among nurses [20]. Nurse managers reported that they have a strong sense of professional pride and a strong sense of mission [14,16,21]. Chinese nurse managers recognised the importance of nursing work during the crisis [14]. They consistently demonstrated positive role-modelling (“Leaders must set a good example and take the lead in communicating with patients.” [15]).

**Theme 2: Dual pressure from resources and the environment**

This theme addresses the critical external constraints that significantly impacted the operational landscape for nurse managers. It emphasizes how inadequacies in tangible resources and the physical work environment exacerbated managerial challenges.

1. Resources shortages

Nurse managers frequently cited shortages in medical staff and protective equipment [16,17,19,23].

Fear of infection, curfews, psychological issues, remote locations and transport difficulties led to increased nurse absenteeism and workforce shortages. In South Africa, 55% of rural nurses were unable to report to work due to curfews [24]. Nurse managers faced the efficiency dilemma of ‘getting familiar with the job and then being rotated’ [14]. Along with the issue of high staff turnover, with numerous nursing positions remaining unfilled for extended periods (“There are many positions for all nursing categories that are supposed to be advertised and filled and they have been there for a very long time.” [19]).

A shortage of protective gear, including clothing and masks, was prevalent, with distribution being uneven. General wards were systematically overlooked, sometimes receiving no PPE, which was instead distributed solely to maternity and COVID-19 wards [19]. Australian nurses had resorted to modifying diving goggles as a substitute for protective equipment, with some using diving masks adapted with HEPA filter connectors [21].

2. Environment deficiencies

Nurse managers encounter physical and environmental limitations, making it challenging to multiplicate management load.

The pandemic prevention and control measures, such as universal screening of staff and visitors, documenting activities, and tracing close contacts, substantially augmented the administrative workload, complicating the adaptation to operational changes [18,19]. Additionally, they were required to maintain constant availability to support their staff, essentially being ‘present’ around the clock [20]. In order to put in place procedures governing the conduct of medical personnel during the pandemic, nurse managers had to recognize their wards at short notice (“There’s simply no room for them here. With its limited capacity, well, the ward is what it is. We won’t move a wall or build anything, you know” [21,24]).

**Theme 3: Core Competency Requirements**

This theme identifies and groups together the essential capabilities that nurse managers need to perform effectively in crisis situations. It goes beyond routine management skills to outline the dynamic competencies required to lead teams, ensure safety, and maintain care quality amid uncertainty.

1. Ongoing training

Whether it is nursing managers themselves or nurses, continuing education and practical training are priorities during EIDs [14]. Nurse managers’ professional skills are under scrutiny, and there is a need to update their knowledge in infectious disease prevention and control [21]. In addition, they confronted the dual challenges of managing a transient staff and quickly adapting to novel operational contexts [20]. They also provided ongoing training for nurses. Chinese nurse managers dedicated half a day to retraining each session, ensuring each individual was thoroughly checked until they were confident [15]. In South Africa, ‘frequent training in donning and doffing’ was mandated [24].

2. Crisis Leadership

In the context of EIDs caring, nurse managers face multiple challenges in exercising crisis leadership. Information chaos was a key obstacle to decision-making, with delayed or contradictory information and frequent policy shifts plunging managers into a state of ‘decision doubt’ [17,22]. They reported utilizing their professional expertise to prioritize urgent matters and make swift decisions amidst information scarcity (“Nobody could possibly follow the rule because nobody knew what the rule was.” [16]).

In crises or prolonged emergencies, decision-making requires swift adaptation to limited or fluid information, demanding more flexible, context-tailored leadership. To cope with the urgent and swiftly changing government directives, nurse managers in the UK had embraced a hierarchical ‘command-and-control’ and transactional leadership approach [22].

The EIDs treatment zone, characterized by heavy workloads and technical complexities, served as a high-pressure testing ground for nurse managers’ administrative competence and professional proficiency [14,20]. It also challenged their ability to conduct rapid risk assessments [17,22].

3. Multiple roles in the team

Nurse managers assumed multifaceted roles within their teams during the care of patients with EIDs. Many respondents emphasized their role as ‘psychotherapists’, providing reassurance and education to alleviate staff anxieties and help them maintain professional composure [15] (“My priority with the staff was to make sure they didn’t lack anything; to listen to them in case someone didn’t feel well enough to work; to talk to them to give them more days off if they needed it, or to replace them with other people, so that they would be well, calm, not overwhelmed.” [18]).

Beyond providing psychological support, nurse managers are responsible for ensuring the safety and well-being of their staff. They ensure that nurses receive adequate rest breaks and are properly equipped with personal protective equipment, thereby minimizing the risk of infection during patient care [18]. Additionally, they reported offering insurance benefits and organizing resilience training programs. Managers boost nurses’ professional effectiveness and demonstrate commitment to their long-term career sustainability [23].

4. Communication and collaboration skills

During the pandemic, physicians and nurses reduced direct patient contact, resulting in communication breakdowns, role conflicts, and increased stigma [10,21]. There had been a lack of proper communication between doctors and nurses (“And then there was this conflict between the doctors and the nurses. I think it got even worse… ethical or not, but it did get worse.” [21]).

Nurse managers reported that they had improved their communication skills and fostered harmonious internal relationships. Leveraging digital platforms such as daily briefings and video conferences can help standardize policies and strengthen inter-team communication [18,20]. Meanwhile, virtual remote visits served as a tool for developing communication strategies with patients’ families [23,25].

**Theme 4: Organizational and system support**

This theme underscores the indispensable role of higher-level organizational structures and leadership in enabling nurse managers to fulfill their responsibilities. It shifts the focus from individual competencies to the systemic support required to sustain them.

1. Strategic reserves

Hospitals have bolstered pandemic preparedness through systematic human and material resource mobilization. On the material front, this involves deploying trained rapid-response teams, establishing segregated triage zones for suspected and confirmed cases, equipping ambulances with disinfection supplies, and implementing spatial renovation, including isolation wards, dedicated clean pathways, and medical equipment upgrades [21,23,24]. Staff welfare provisions like disposable attire, rest areas, and bathing facilities are also prioritized to support operational sustainability [21].

Furthermore, nurse managers reported that hospitals optimized resource allocation by leveraging medical staff expertise and developing a tiered treatment system via team restructuring, retraining, and fostering interdepartmental collaboration (“We established teams where there was an RN team lead, and that had to be a regular orthopedic nurse who was the team lead for these medical patients.” [25].

2. Superiors’ support

Nurse managers expressed significant dissatisfaction with the lack of support from their superiors. When hospital management decisions were communicated to staff through them, they often exacerbated the already dire situation in the wards (“Even if I tell others that there is a lack of support here and that I feel like I’m fighting alone on the front lines, it doesn’t help, because management has no intention of changing the status quo” [21]). This sense of powerlessness stemmed from a breakdown in communication between upper and lower levels. It highlighted the voiceless state of frontline managers within the decision-making process [22].

Ward managers, in particular, felt marginalized by hospital leadership. Despite bearing direct responsibility for managing healthcare staff and patients, they were excluded from decision-making regarding the pandemic. They criticized the top-down information dissemination model whereby ‘nurse managers were often excluded from high-level meetings’ [20].

Nurse managers urgently required substantive support from senior management, including assistance with specific tasks such as setting up nurse workstations [21]. This demand highlighted the breakdown in the support chain within the management structure during the pandemic.

Future support and development efforts have recognized the necessity for a structured methodology in cultivating strategic-level nurse leaders, complete with chances to gain hands on experience and learning at this leadership tier (“Thinking about how you can help leaders, nursing leaders and midwifery leaders of the future. Go look, see.” [22]).

## 4. Discussion

This meta-synthesis systematically analyzed 13 qualitative studies to reveal nurse managers’ multifaceted experiences and competency development needs during EID outbreaks. Evidence from the included studies demonstrates that nurse managers face diverse challenges during such crises, broadly categorized as multidimensional experiences, dual pressure from resources and the environment, core competency requirements and organizational and system support.

During the outbreak of EIDs, nurse managers face various problems, including severe shortages of personnel and supplies, difficulties in emergency management, obstacles to team collaboration and leadership, and a notable increase in workload [10,16,17,19,21,23]. According to the JD-R model, these compounding demands outstrip available organizational resources [27], which may explain why nurse managers have reported burnout and a decline in personal fulfilment [19,22]. Consistent with COR theory, the cumulative loss of resources—including sleep deprivation, reduced social support and a diminished sense of control—may trigger a stress response and exacerbate anxiety and role conflict [28]. These experiences often involve intertwined physical and psychological burdens, with negative impacts predominating [17,19,20,21]. These manifestations align with Ahlqvist’s description of the physical and psychological toll of resource depletion [29]. Nurse managers are not only assigned the responsibility of dealing with crises operationally, but also mentally, emotionally, and even morally [30]. A study in Spain also showed a significant increase in perceived stress among nurse managers during the COVID-19 pandemic [31]. Our study identifies widespread detrimental psychological experiences, notably emotional exhaustion and pervasive insecurity, chronic anxiety, role conflict, work–life imbalance, discrimination, and workplace bullying [10]. These adverse experiences may precipitate burnout [32], reduce job satisfaction [33], and prompt nurse managers to occasionally or more seriously consider other job and career options [29], and even resignation [34].

These findings regarding resource shortages and workforce pressures are consistent with studies from Iran and Canada, which reported that infections and deaths among nursing staff placed additional strain on hospitals and exacerbated nursing shortages during the COVID-19 crisis [35,36]. As our meta-synthesis highlights, these pressures increase the workload and leadership challenges faced by nurse managers and may indirectly affect clinical outcomes [37]. Despite these challenges, nurse managers also reported positive outcomes: strengthened professional identity, sense of accomplishment, collective pride, personal growth, team cohesion, and deeper appreciation of their vocation [14,15,17,20,21,22]. Several studies [38,39,40] attribute these outcomes to target organizational practices such as weekly peer-support debriefings, crisis leadership workshops, and mentorship pairing programs, which foster team cohesion and reframe challenges as collective achievements. As in Sanabria-Delgado et al. [41], nurse managers’ positive reflection and optimism emerge in the midst of a relentless struggle. The best will come from the worst because you learn from everything.

Nurse managers undertake and support frontline nursing staff in completing their work, reducing their psychological burden and stress [14]. Addressing lingering psychological impacts is crucial. Providing nurse managers with robust organizational support and psychological resources is not only critical for their personal health and well-being, but also essential for ensuring the robust functioning of the nursing system. Research by Middleton et al. [33] demonstrates that additional organizational support is critical to enable nurse managers to implement coping strategies aimed at safeguarding their physical and psychological well-being. An American study showed that social support and self-care are important strategies for resilience reported by nurse managers [42]. To effectively mitigate emotional distress and foster long-term resilience, we recommend integrating free counseling services from employee assistance programs into routine organizational practices, while concurrently embedding evidence-based coping mechanism training within daily workflows. Complementing this, occupational safeguards need to be actively developed and enforced. This involves conducting regular psychosocial risk assessments, implementing practical measures to improve work–life balance, ensuring fair and supportive management practices.

During the early stages of EIDs, nurse managers also face the particular challenge of constant communication. Difficulties in information flow, frequent updates to guidelines and prevention protocols lead to uncertainty and decision-making confusion. This is consistent with Pelli et al. [43], who identify communication breakdowns as inducing frustration among nursing managers during EIDs.

Our meta-synthesis further indicates that the involvement of nurses in policy-making remains limited overall [44,45], resulting in information gaps and policy ambiguities that hinder implementation. Policy-makers and decision-makers should heed the experiences and voices of nurses, and organizations should embed nurse managers in emergency governance structures [46]. This ensures frontline insights inform strategic planning. Furthermore, strengthening crisis leadership training by focusing on interactive communication, active networking, and change management is critical. A Finnish study [47] of nurse managers during COVID-19 identifies these exact competencies as indispensable for effective crisis response, and a web-based crisis management program markedly improves participants’ crisis-management, care-service management, and leadership skills post-training [47,48]. EIDs have catalyzed a global shift in the professional landscape of hospital nurse managers, as contemporary challenges now require competencies that transcend traditional management frameworks to address evolving clinical, organizational, and ethical complexities. There is a pressing need to provide nurse managers with structured training in interactive communication, active networking, and change management to update their competencies so that they will be able to manage crisis situations effectively in the future [10,47,49].

Nurse managers in our meta-synthesis cite a shortage of workforce and a lack of cohesion in newly formed teams, leading to increased complexity for managers. A German study [50] shows the demand for nursing care is escalating, while there is a concurrent strain on the availability of specialized nursing workforce. The findings highlight the need for measures to ensure the provision of long-term care, especially in times of such crises. A Chinese study [51] shows that scheduling nursing workforce to meet the demands of epidemic relief is a key issue facing nurse managers in healthcare facilities today.

Several methodological limitations of this meta-synthesis should be acknowledged. All 13 studies focused specifically on the COVID-19 pandemic, reflecting the global research priorities during the crisis. Whilst the synthesised themes provide valuable insights into the experiences of nurse managers during the pandemic, it remains uncertain whether these findings can be generalised to the contexts of other emerging infectious diseases such as Ebola, SARS or MERS. Each emerging infectious disease has its own unique transmission dynamics, mortality rates and public health response measures, factors which may influence the challenges and competency requirements faced by nurse managers in different ways. Consequently, the findings of this study should primarily be regarded as evidence derived from the COVID-19 pandemic, and caution should be exercised when extrapolating them to other emerging infectious diseases. Furthermore, our restriction to English- and Chinese-language literature may have introduced language and publication biases, as relevant qualitative studies published in other languages may have been omitted, thereby limiting the transferability of our findings to healthcare systems in countries where English or Chinese is not the primary language Additionally, the included studies employed a variety of job titles for nurse managers, such as “ward manager”, “nurse practitioner” and “chief nursing officer”. These variations in roles and responsibilities across healthcare systems may affect the conceptual consistency of our synthesised findings. Furthermore, most original studies lacked explicit statements regarding reflexivity and the researchers’ stance. This is reflected in JBI evaluation dimensions Q6 and Q7, which may affect the interpretative credibility of both the original studies and the synthesis. Another limitation is the heterogeneity of qualitative methods across the included studies; we did not systematically explore how this methodological diversity might influence the synthesised results. Finally, we did not employ a formal confidence assessment framework, such as GRADE-CERQual or ConQual, to assess confidence in the synthesised qualitative findings. Such an assessment would have provided readers with a clearer indication of the level of confidence for each theme. Future research should address these limitations by expanding language coverage, standardising role definitions, incorporating reflective practice, and applying confidence assessment tools.

## 5. Conclusions

Our meta-synthesis synthesizes the lived experiences and core competency requirements of nurse managers during EID outbreaks. The findings indicate that nurse managers confront a multitude of challenges, including considerable psychological distress and resource limitations, while concurrently exhibiting resilience and leadership development. These experiences underscore the critical need for robust organizational support systems. Concurrently, the identified competencies, such as crisis leadership, effective communication, and adaptive decision-making, provide a clear framework for targeted training and development. However, as all the evidence relates to COVID-19, its applicability to other EIDs may be limited. Consequently, future endeavors should prioritize the development of integrated, evidence-based training programs that combine technical, emotional, and systemic competencies. These programs should aim to equip nurse managers with the skills necessary to function effectively in high-pressure environments, thereby enhancing the resilience of healthcare systems and improving patient outcomes. For future research, longitudinal and multi-site studies are needed to examine how nurse managers‘ competencies evolve across different phases of infectious disease outbreaks. Comparative studies across diverse healthcare systems and cultural contexts would also help clarify how organizational structures shape nurse managers’ experiences and leadership practices. Additionally, future reviews should include studies published in a broader range of languages and apply formal confidence assessment frameworks, such as GRADE-CERQual or ConQual, to strengthen the credibility of synthesized findings.

### Implications for Nursing and Health Policy

While the evidence for this synthesis derives exclusively from the COVID-19 pandemic, the suddenness and complexity of EIDs more broadly demonstrate that the challenges faced by nurse managers in practice and the gaps in their capabilities are key areas of focus for optimising nursing practice and refining policy. At the policy level, “institutional guarantees” should be regarded as the key: endow nurse managers with discourse power in collaboration by clarifying the boundaries of their powers and responsibilities, resolve their resource shortages through regular resource supply, and break down inter-departmental barriers by means of standardized collaboration mechanisms, thus allowing nurse managers to “have the conditions” to play their role as a hub. Ultimately, this will achieve the multiple guarantees of patient safety, nurses’ rights and interests, and public health security.

## Figures and Tables

**Table 1:** Assessment of methodological quality.

Study	Q1	Q2	Q3	Q4	Q5	Q6	Q7	Q8	Q9	Q10	Level
Shi et al. (2020) [14]	U	Y	Y	Y	Y	Y	Y	Y	U	Y	B
Cheng et al. (2020) [15]	U	Y	Y	Y	Y	U	U	Y	U	Y	B
Monroe et al. (2022) [16]	Y	Y	Y	Y	Y	U	U	Y	Y	Y	B
Riddell et al. (2022) [17]	Y	Y	Y	Y	Y	U	U	Y	Y	Y	B
Vázquez-Calatayud et al. (2022) [18]	U	Y	Y	Y	Y	Y	U	Y	Y	Y	B
Moyo et al. (2022) [19]	Y	Y	Y	Y	Y	U	U	Y	Y	Y	B
Holge-Hazelton et al. (2021) [20]	U	Y	Y	Y	Y	Y	U	Y	Y	Y	B
Dobrowolska et al. (2023) [21]	U	Y	Y	Y	Y	U	U	Y	Y	Y	B
James et al. (2025) [22]	Y	Y	Y	Y	Y	U	U	Y	Y	Y	B
Ahmed et al. (2022) [23]	Y	Y	Y	Y	Y	N	N	Y	Y	Y	B
Hossny et al. (2022) [24]	U	Y	Y	Y	Y	U	U	Y	Y	Y	B
Langan et al. (2022) [25]	U	Y	Y	Y	Y	N	N	Y	Y	Y	B
Chen et al. (2023) [10]	Y	Y	Y	Y	Y	N	N	Y	Y	Y	B

Note: Aromataris E, Munn Z (Editors). JBI Manual for Evidence Synthesis. JBI, 2020. Available from https://synthesismanual.jbi.global (accessed on 15 April 2025). https://doi.org/10.46658/JBIMES-20-01 [26]. Q1 Is there congruity between the stated philosophical perspective and the research methodology?; Q2 Is there congruity between the research methodology and the research question or objectives?; Q3 Is there congruity between the research methodology and the methods used to collect data?; Q4 Is there congruity between the research methodology and the representation and analysis of data?; Q5 Is there congruity between the research methodology and the interpretation of results?; Q6 Is there a statement locating the researcher culturally or theoretically?; Q7 Is the influence of the researcher on the research, and vice versa, addressed?; Q8 Are participants, and their voices, adequately represented?; Q9 Is the research ethical according to current criteria or, for recent studies, and is there evidence of ethical approval by an appropriate body?; Q10 Do the conclusions drawn in the research report flow from the analysis, or interpretation, of the data? Y = Yes; N = No; U = Unclear; B = Grade B (5–8 points).

**Table 2:** Demographic characteristics of the participants of the individual studies included in the thematic synthesis.

Author/Year	Country	Research Design	Data Collection	Study Subject	Hospital
Shi et al. (2020) [14]	China	Descriptive phenomenology	Semi-structured interviews	11 nurse managers	Tertiary general hospital, Wuhan
Cheng et al. (2020) [15]	China	Qualitative	Semi-structured interviews	8 nurse managers	Jiaxing First Hospital, Zhejiang
Monroe et al. (2022) [16]	USA	Descriptive qualitative	Semi-structured interviews	9 nurse managers	Hospitals across the U.S.
Riddell et al. (2022) [17]	Australia	Constructivist qualitative	Semi-structured interviews	14 nurse managers	hospitals, Melbourne
Vázquez-Calatayud et al. (2022) [18]	Spain	Descriptive phenomenology	Semi-structured interviews	10 nurse managers	university hospital, Pamplona
Moyo et al. (2022) [19]	South Africa	Descriptive phenomenology	In-depth unstructured telephone interviews	10 nurse managers	District hospital in Limpopo Province
Holge-Hazelton et al. (2021) [20]	Denmark	Qualitative directed content analysis	Telephone interviews	13 nurse managers	University Hospital
Dobrowolska et al. (2023) [21]	Poland	Qualitative phenomenology	Phone interviews	15 nurse managers	Multiple hospitals across Poland
James et al. (2025) [22]	UK	Qualitative	Online semi-structured interviews	21 nurse managers	NHS Trusts/Health Boards
Ahmed et al. (2022) [23]	United Arab Emirates	Descriptive qualitative	Semi-structured interviews	37 nurse managers	13 governmental/non-governmental hospitals;
Hossny et al. (2022) [24]	Egypt	Qualitative content analysis	Semi-structured interviews	35 nurse managers	5 university hospitals
Langan et al. (2022) [25]	USA	Grounded theory + Content analysis	Semi-structured telephone interviews	13 nurse managers	Hospital not specified
Chen et al. (2023) [10]	China	Descriptive Phenomenology	Face-to-face semi-structured interviews	18 nurse managers	3 municipal hospitals

**Table 3:** Results of thematic synthesis.

Main Theme	Subthemes	Findings
Theme 1: Multidimensional experiences of nurse managers	Dual physical and mental burden	Physical exhaustion
		Emotional exhaustion
		Concern
		PTSD
		Moral dilemma
		Role conflict
	Social trauma	Workplace exclusion
		New manager workload
		Community stigmatization challenges
	Positive attitude	Growing through adversity
		Sense of achievement
		Collaborative empowerment
		Professional identity
		Professional honor
Theme 2: Dual pressure from resources and the environment	Resources shortages	Human resource collapse
		Lack of protective equipment
	Environment deficiencies	Pressure of epidemic prevention and control
		Physical environmental constraints
Theme 3: Core Competency Requirements	Ongoing training	Professional skills
		Staff training
	Crisis Leadership	Responding to information chaos
		Flexible leadership strategies
		High-pressure capability tests
	Multiple roles in the team	Psychological counseling
		Safety and security
		Team empowerment
	Communication and collaboration skills	Medical communication
		Patient–caregiver communication
Theme 4: Organizational and system support	Strategic reserves	Material resource reserves
		Human resource optimization
		Cross-departmental collaboration strategies
	Superiors’ support	Lack of support
		Insufficient participation in decision-making
		Substantive support demands
		Leadership development program

## Data Availability

Data sharing is not applicable to this article as no datasets were generated or analyzed during the current study.

## Supplementary Materials

The following supporting information can be downloaded at https://www.mdpi.com/article/10.3390/healthcare14142088/s1, File S1: Search Strategies. File S2: PRISMA 2020 Checklist. Reference [52] is included in the Supplementary Materials.

## References

[1] Wang M., Yang B., Liu Y., Yang Y., Ji H., Yang C. (2023). Emerging infectious disease surveillance using a hierarchical diagnosis model and the Knox algorithm. Sci. Rep..

[2] Zhang L., Li M.Y., Zhi C., Zhu M., Ma H. (2024). Identification of Early Warning Signals of Infectious Diseases in Hospitals by Integrating Clinical Treatment and Disease Prevention. Curr. Med. Sci..

[3] Catania G., Zanini M., Hayter M., Timmins F., Dasso N., Ottonello G., Aleo G., Sasso L., Bagnasco A. (2021). Lessons from Italian front-line nurses’ experiences during the COVID-19 pandemic: A qualitative descriptive study. J. Nurs. Manag..

[4] Cummings G.G., Lee S., Tate K., Penconek T., Micaroni S.P.M., Paananen T., Chatterjee G.E. (2021). The Essentials of Nursing Leadership: A Systematic Review of Factors and Educational Interventions Influencing Nursing Leadership. Int. J. Nurs. Stud..

[5] Kanter R.M. (1977). Men and Women of the Corporation.

[6] Hofmeyer A., Taylor R. (2021). Strategies and Resources for Nurse Leaders to Use to Lead with Empathy and Prudence so They Understand and Address Sources of Anxiety Among Nurses Practising in the Era of COVID-19. J. Clin. Nurs..

[7] Galehdar N., Kamran A., Toulabi T., Heydari H. (2020). Exploring Nurses’ Experiences of Psychological Distress During Care of Patients with COVID-19: A Qualitative Study. BMC Psychiatry.

[8] Perraud F., Ecarnot F., Loiseau M., Laurent A., Fournier A., Lheureux F., Binquet C., Rigaud J.P., Meunier-Beillard N., Quenot J.P. (2022). A Qualitative Study of Reinforcement Workers’ Perceptions and Experiences of Working in Intensive Care During the COVID-19 Pandemic: A Psycovid-ICU Substudy. PLoS ONE.

[9] HøLGE-Hazelton B., Kjerholt M., Rosted E., Thestrup Hansen S., Zacho Borre L., McCormack B. (2021). Health Professional Frontline Leaders’ Experiences During the COVID-19 Pandemic: A Cross-Sectional Study. J. Healthc. Leadersh..

[10] Chen Y., Jiang H., Shen Y., Zhou P. (2023). Nurse Managers’ Experience During the COVID-19 Pandemic In China: A Qualitative Study. Nurs. Open.

[11] Lockwood C., Munn Z., Porritt K. (2015). Qualitative Research Synthesis: Methodological Guidance for Systematic Reviewers Utilizing Meta-Aggregation. JBI Evid. Implement..

[12] Dickinson N., Spencer L.H., Yang S., Miller C., Hursthouse A., Lynch M. (2025). Extreme weather events in the UK and resulting public health outcomes. Int. J. Public Health.

[13] Thomas J., Harden A. (2008). Methods for the Thematic Synthesis of Qualitative Research in Systematic Reviews. BMC Med. Res. Methodol..

[14] Shi Y.Q., Dai X.J., Feng Z.C. (2020). Qualitative Research on Working Experience of Head Nurses in COVlD-19 Wards in General Hospitals. J. Nurs..

[15] Cheng J.Y., Yao M., Xu X.Y., Ju Q.Y., Zhang M.Q., Fei Y.P., Yang D.M., Ma J.M., Zhang H.Y., Wang Y.C. (2020). A Retrospective Study of the Management Work Experience in an Isolation Ward for Novel Coronavirus Pneumonia. Chin. J. Integr. Tradit. West. Med. Intensive Crit. Care.

[16] Monroe M., Davies C.C., Beckman D., Cantrell D., Brockopp D. (2022). Chief Nursing Officers Their COVID-19 Experience. J. Nurs. Adm..

[17] Riddell K., Bignell L., Bourne D., Boyd L., Crowe S., Cucanic S., Flynn M., Gillan K., Heinjus D., Mathieson J. (2022). The Context, Contribution and Consequences of Ad-dressing the COVID-19 Pandemic: A Qualitative Exploration of Executive Nurses’ Perspectives. J. Adv. Nurs..

[18] Vazquez-Calatayud M., Regaira-Martinez E., Rumeu-Casares C., Aloma-Mora B., Esain A., Oroviogoicoechea C. (2022). Experiences of Frontline Nurse Managers During the COVID-19: A Qualitative Study. J. Nurs. Manag..

[19] Moyo I., Mgolozeli S.E., Risenga P.R., Mboweni S.H., Tshivhase L., Mudau T.S., Ndou N.D., Mavhandu-Mudzusi A.H. (2022). Experiences of Nurse Managers during the COVID-19 Outbreak in a Selected District Hospital in Limpopo Province, South Africa. Healthcare.

[20] Holge-Hazelton B., Kjerholt M., Rosted E., Thestrup Hansen S., Zacho Borre L., McCormack B. (2021). Improving Person-Centred Leadership: A Qualitative Study of Ward Managers’ Experiences During the COVID-19 Crisis. Risk Manag. Healthc. Policy.

[21] Dobrowolska B., Gutysz-Wojnicka A., Dziurka M., Ozdoba P., Ozga D., Penar-Zadarko B., Markiewicz R., Markiewicz-Gospodarek A., Palese A. (2023). Intensive Care Nurse Managers’ Experiences During the First Wave of the COVID-19 Pandemic: Implications for Future Epidemiological Crises. PLoS ONE.

[22] James A.H., Dimond R., Jones A., Watkins D., Kelly D. (2025). Leading through the COVID-19 pandemic: Experiences of UK Executive Nurse Directors. J. Adv. Nurs..

[23] Ahmed F.R., Dias J.M., Al Yateem N., Subu M.A., Abu Ruz M. (2022). Lessons Learned and Recommendations from the COVID-19 Pandemic: Content Analysis of Semi-Structured Interviews with Intensive Care Unit Nurse Man-agers in the United Arab Emirates. J. Nurs. Manag..

[24] Hossny E.K., Morsy S.M., Ahmed A.M., Saleh M.S.M., Alenezi A., Sorour M.S. (2022). Management of the COVID-19 Pandemic: Chal-lenges, Practices, and Organizational Support. BMC Nurs..

[25] Langan J.C., Griffin A.R., Shipman S., Dobalian A.l. (2022). Nurse Executive Experiences with COVID-19: Now We Know-We Are Not Going Back. Nurs. Adm. Q..

[26] Aromataris E., Munn Z. (2020). JBI Manual for Evidence Synthesis.

[27] Bakker A.B., Demerouti E. (2024). Job Demands-Resources Theory: Frequently Asked Questions. J. Occup. Health Psychol..

[28] Albo Y., Leck E., Nathan O., Wolf N., Zaidise E. (2025). Cor and Corona: Analysis Of COVID-19’s Subjective Lasting Impact on Wellbeing, Employing Conservation of Resources Theory. J. Public Health.

[29] Ahlqvist A., Pursio K., Nurmeksela A., Kvist T. (2025). Nurse Managers’ Experiences of Work Well-Being During the COVID-19 Pandemic. BMC Nurs..

[30] Dimino K., Learmonth A.E., Fajardo C.C. (2021). Nurse Managers Leading the Way: Reenvisioning Stress to Maintain Healthy Work Environments. Crit. Care Nurse.

[31] Boned-Galan A., Lopez-Ibort N., Gil-Lacruz A.I., Gascón-Catalán A. (2023). Stress Impact of COVID-19 in Nurse Managers. Heliyon.

[32] Dall’ora C., Ball J., Reinius M., Griffiths P. (2020). Burnout in Nursing: A Theoretical Review. Hum. Resour. Health.

[33] Hsu H.C., Lee H.F., Hung H.M., Chen Y.L., Yen M., Chiang H.Y., Chow L.H., Fetzer S.J., Mu P.F. (2024). Effectiveness of Individual-Based Strategies to Reduce Nurse Burnout: An Umbrella Review. J. Nurs. Manag..

[34] Middleton R., Loveday C., Hobbs C., Almasi E., Moxham L., Green H., Halcomb E., Fernandez R. (2021). The COVID-19 Pandemic—A Focus on Nurse Managers’ Mental Health, Coping Behaviours and Organisational Commitment. Collegian.

[35] Heydarikhayat N., Ghanbarzehi N., Darban F., Kashani Z.A., Rohani C. (2024). Exploring Lived Experiences of Vulnerability in Nursing Management during the Coronavirus Disease 2019 Pandemic: A Phenomenological Study of Nurse Managers and Nurses. SAGE Open Nurs..

[36] Udod S., Baxter P., Gagnon S., Halas G., Raja S. (2024). Experiences of Frontline Managers during the COVID-19 Pandemic: Recommendations for Organizational Resilience. Healthcare.

[37] Li L.Z., Yang P., Singer S.J., Pfeffer J., Mathur M.B., Shanafelt T. (2024). Nurse Burnout and Patient Safety, Satisfaction, and Quality of Care: A Systematic Review and Meta-Analysis. JAMA Netw. Open.

[38] Peng X., Yang Y., Gao P., Ren Y., Hu D., He Q. (2022). Negative and Positive Psychological Experience of Frontline Nurses in Combatting COVID-19: A Qualitative Study. J. Nurs. Manag..

[39] Foli K.J., Forster A., Cheng C., Zhang L., Chiu Y.C. (2021). Voices From the COVID-19 Frontline: Nurses’ Trauma and Coping. J. Adv. Nurs..

[40] Thrysoee L., Dyrehave C., Christensen H.M., Jensen N.B., Nielsen D.S. (2022). Hospital Nurses’ Experiences of and Perspectives on the Impact COVID-19 Had on Their Professional and Everyday Life-a Qualitative Interview Study. Nurs. Open.

[41] Sanabria-Delgado D., Barrientos-Trigo S., PORCEL-Gálvez A.M. (2025). Exploring the Long-Term Impact of Emotional Exhaustion on Frontline Nurse Managers Post-COVID-19: A Qualitative Study. J. Nurs. Manag..

[42] Montgomery A.P., Patrician P.A. (2024). COVID-19 Stressors and Resilience Among Nurse Leaders. Nurs. Adm. Q..

[43] Pelli J., Nordquist H. (2023). Learning Lessons for Future Preparedness: Exploring Work Well-Being-Related Leadership Challenges among Paramedics during the Early Stage of the COVID-19 Pandemic-A Qualitative Study. Nurs. Rep..

[44] Rasheed S.P., Younas A., Mehdi F. (2020). Challenges, Extent of Involvement, and the Impact of Nurs-es’ Involvement in Politics and Policy Making in in Last Two Decades: An Integrative Review. J. Nurs. Scholarsh..

[45] Al Yahyaei A., Al Rasbi S., Al Kindi Z., Al Omari O., Al Sabei S., Al Hashmi N., Al Balushi N. (2025). Empowering voices: Predictors and levels of nurses’ involvement in healthcare policy. Int. Nurs. Rev..

[46] Luo Y., Feng X., Wang D., Qiao X., Xiao X., Jia S., Zheng M., Reinhardt J.D. (2023). Experience of Clinical Nurses Engaged in Caring for Patients with COVID-19: A Qualitative Systematic Review and Meta-Synthesis. J. Clin. Nurs..

[47] Jääski T., Talvio H., Kuha S., Kanste O. (2024). Crisis Management Competencies Needed in a Hospital Setting During the COVID-19 Pandemic: A Qualitative Study of Nurse Leaders. Nurs. Open.

[48] Alan H., Harmanci Seren A.K., Eskin Bacaksiz F., Güngör S., Bilgin O., Baykal Ü. (2023). An Evaluation of a Web-Based Crisis Management Training Program for Nurse Managers: The Case of the COVID-19 Crisis. Disaster Med. Public Health Prep..

[49] Filomeno L., Feller E.A., Raimondi F., Di Mario S. (2024). Nurse Managers Coping Strategies for Crisis Management: Qualitative Systematic Review. Enferm. Clin. Engl. Ed..

[50] Pförtner T.-K., Pfaff H., Hower K.I. (2021). Will the demands by the COVID-19 pandemic increase the intent to quit the profession of long-term care managers: A repeated cross-sectional study in Germany. J. Public Health.

[51] Liu X.N., Xiao M.Z., Wang D.R., Zhao Q.H., Xu W.X. (2021). Current Status of Nurse Staffing: A Qualitative Study Based on Nursing Manager’s Perspective. J. Third Mil. Med. Univ..

[52] Page M.J., McKenzie J.E., Bossuyt P.M., Boutron I., Hoffmann T.C., Mulrow C.D., Shamseer L., Tetzlaff J.M., Akl E.A., Brennan S.E. (2021). The PRISMA 2020 statement: An updated guideline for reporting systematic reviews. BMJ.

