# Supplementary material for: Nurse Managers’ Experiences and Competency Requirements in the Care of Patients with Emerging Infectious Diseases: A Meta-Synthesis
Source: Healthcare (Basel). 2026 Jul 13;14(14):2088. doi: 10.3390/healthcare14142088 (PMC13412099; doi:10.3390/healthcare14142088)
Supplement: Supplementary file 1 [file healthcare-14-02088-s001.zip › Supplementary File S1. Search Strategies.pdf]

Supplemental File 3: Search strategies

Search strategies used for finding qualitative research articles about nurse managers' experiences and competency requirements in the care of patients with Emerging infectious diseases. Number of retrieved articles is given in the right-hand column.

PUBMED		
1#	(((((((((((Nurse Administrators[MeSH Terms]) OR (Administrator, Nurse[Title/Abstract])) OR (Administrators, Nurse[Title/Abstract])) OR (Nurse Administrator[Title/Abstract])) OR (Nurse Managers[Title/Abstract])) OR (Manager, Nurse[Title/Abstract])) OR (Managers, Nurse[Title/Abstract])) OR (Nurse Manager[Title/Abstract])) OR (Nurse Executives[Title/Abstract])) OR (Executive, Nurse[Title/Abstract])) OR (Executives, Nurse[Title/Abstract])) OR (Nurse Executive[Title/Abstract])	17642
2#	((((((((Communicable Diseases, Emerging[MeSH Terms]) OR (Severe acute respiratory syndrome[MeSH Terms])) OR (Middle East Respiratory Syndrome Coronavirus[MeSH Terms])) OR (Hemorrhagic Fever, Ebola[MeSH Terms])) OR (Marburg Virus Disease[MeSH Terms])) OR (Lassa Fever[MeSH Terms])) OR (Nipah Virus[MeSH Terms])) OR (Zika Virus Infection[MeSH Terms])) OR (COVID-19[MeSH Terms])) OR (Communicable Disease, Emerging[Title/Abstract])) OR (Disease*, Emerging Communicable[Title/Abstract])) OR (Emerging Communicable Disease*[Title/Abstract])) OR (Infectious Diseases, Emerging[Title/Abstract])) OR (Disease*, Emerging Infectious[Title/Abstract])) OR (Emerging Infectious Disease*[Title/Abstract])) OR (Infectious Disease, Emerging[Title/Abstract]))) OR (Respiratory Syndrome, Severe Acute[Title/Abstract])) OR (SARS[Title/Abstract])) OR (Respiratory Syndrome, Acute, Severe[Title/Abstract])) OR (MERS Virus*[Title/Abstract])) OR (Virus, MERS[Title/Abstract])) OR (Middle East respiratory syndrome-related coronavirus[Title/Abstract])) OR (Middle East respiratory syndrome related coronavirus[Title/Abstract])) OR (MERS-CoV[Title/Abstract])) OR (Merbecovirus*[Title/Abstract])) OR (Ebola Infection[Title/Abstract])) OR (Infection, Ebola[Title/Abstract])) OR (Ebola Virus Disease[Title/Abstract])) OR (Ebola Virus Infection[Title/Abstract])) OR (Infection, Ebola Virus[Title/Abstract])) OR (Virus Infection, Ebola[Title/Abstract])) OR (Ebolavirus Infection*[Title/Abstract])) OR (Infection*, Ebolavirus[Title/Abstract])) OR (Ebola Hemorrhagic Fever[Title/Abstract])) OR (Marburg Hemorrhagic Fever[Title/Abstract])) OR (Fever, Marburg Hemorrhagic[Title/Abstract])) OR (Hemorrhagic Fever, Marburg[Title/Abstract])) OR (Marburg Disease[Title/Abstract])) OR (Disease, Marburg[Title/Abstract])) OR (Fever, Lassa[Title/Abstract])) OR (Lassa Fevers[Title/Abstract])) OR (Lassa Virus Infection[Title/Abstract])) OR (Infection, Lassa Virus[Title/Abstract])) OR (Lassa Virus Infections[Title/Abstract])) OR (Virus Infection, Lassa[Title/Abstract])) OR	492824

	(Nipah Viruses[Title/Abstract])) OR (Virus*, Nipah[Title/Abstract])) OR (Infection, Zika Virus[Title/Abstract])) OR (Virus Infection, Zika[Title/Abstract])) OR (ZikV Infection[Title/Abstract])) OR (Infection, ZikV[Title/Abstract])) OR (Fever, Zika[Title/Abstract])) OR (Zika Virus Disease[Title/Abstract])) OR (Disease, Zika Virus[Title/Abstract])) OR (Virus Disease, Zika[Title/Abstract])) OR (Zika Fever[Title/Abstract])) OR (Congenital Zika Syndrome[Title/Abstract])) OR (Congenital Zika Virus Infection[Title/Abstract])) OR (2019-nCoV Infection*[Title/Abstract])) OR (Infection, 2019-nCoV[Title/Abstract])) OR (SARS-CoV-2 Infection*[Title/Abstract])) OR (Infection, SARS-CoV-2[Title/Abstract])) OR (SARS CoV 2 Infection[Title/Abstract])) OR (2019 Novel Coronavirus Disease[Title/Abstract])) OR (2019 Novel Coronavirus Infection[Title/Abstract])) OR (COVID-19 Virus Infection*[Title/Abstract])) OR (Infection, COVID-19 Virus[Title/Abstract])) OR (Virus Infection, COVID-19[Title/Abstract])) OR (COVID19[Title/Abstract])) OR (Coronavirus Disease 2019[Title/Abstract])) OR (Disease 2019, Coronavirus[Title/Abstract])) OR (Coronavirus Disease-19[Title/Abstract])) OR (Coronavirus Disease 19[Title/Abstract])) OR (Severe Acute Respiratory Syndrome Coronavirus 2 Infection[Title/Abstract])) OR (COVID-19 Virus Disease[Title/Abstract])) OR (COVID 19 Virus Disease[Title/Abstract])) OR (COVID-19 Virus Diseases[Title/Abstract])) OR (Disease, COVID-19 Virus[Title/Abstract])) OR (Virus Disease, COVID-19[Title/Abstract])) OR (SARS Coronavirus 2 Infection[Title/Abstract])) OR (2019-nCoV Disease*[Title/Abstract])) OR (2019 nCoV Disease[Title/Abstract])) OR (Disease, 2019-nCoV[Title/Abstract])) OR (COVID-19 Pandemic*[Title/Abstract])) OR (COVID 19 Pandemic[Title/Abstract])) OR (Pandemic, COVID-19[Title/Abstract]))	
3#	((((((((Interview[Title/Abstract]) OR (discourse[Title/Abstract])) OR (Content analysis[Title/Abstract])) OR (Phenomenol*[Title/Abstract])) OR ((((Qualitative Research[MeSH Terms]) OR (Research, Qualitative[Title/Abstract])) OR (qualitative method[Title/Abstract])) OR (qualitative stud*[Title/Abstract]))) OR (((focus group[MeSH Terms]) OR (Group, Focus[Title/Abstract])) OR (Groups, Focus[Title/Abstract]))) OR ((Grounded Theory[MeSH Terms]) OR (Theory, Grounded[Title/Abstract]))) OR ((((((((Ethnography[MeSH Terms]) OR (Enthnograph*[Title/Abstract])) OR (Cultural Anthropology[Title/Abstract])) OR (Material Culture[Title/Abstract])) OR (Culture, Material[Title/Abstract])) OR (Material Cultures[Title/Abstract])) OR (Ethnography[Title/Abstract])) OR (Ethnographies[Title/Abstract])))	586601
#4	#1 AND #2 AND #3	85
**Embase**		
#1	'Nurse Administrator'/exp	16,511
#2	'Administrator*, Nurse':ab,ti	43
#3	'Nurse Manager*':ab,ti	4,822
#4	'Manager*, Nurse':ab,ti	156
#5	'Nurse Executive*':ab,ti	1,259

#6	'Executive*, Nurse':ab,ti	79
#7	#1 OR #2 OR #3 OR #4 OR #5 OR #6	19,166
#8	'qualitative research'/exp	144,495
#9	'research, qualitative':ab,ti	298
#10	'qualitative method':ab,ti	2,826
#11	'qualitative stud*':ab,ti	95,252
#12	'interview*':ab,ti	641,481
#13	'phenomenol*':ab,ti	42,487
#14	'discourse':ab,ti	21,653
#15	'content analysis':ab,ti	57,470
#16	'focus group'/exp	37
#17	'grounded theory'/exp	11,684
#18	'ethnography'/exp	4,426
#19	'focus group':ab,ti	49.019
#20	'group, focus':ab,ti	205
#21	'groups, focus':ab,ti	308
#22	'theory, grounded':ab,ti	105
#23	'enthnograph*':ab,ti	0
#24	'cultural anthropology':ab,ti	225
#25	'culture, material':ab,ti	507
#26	'material culture*':ab,ti	619
#27	'ethnograph*':ab,ti	16,473
#28	#8 OR #9 OR #10 OR #11 OR #12 OR #13 OR #14 OR #15 OR #16 OR #17 OR #18 OR #19 OR #20 OR #21 OR #22 OR #23 OR #24 OR #25 OR #26 OR #27	796,877
#29	'Emerging Infectious Disease '/exp	43
	#29 OR #38 OR #39 OR #40 OR #41 OR #42 OR #43 OR #44	6021
#30	'Severe acute respiratory syndrome '/exp	11,863
	#30 OR #45 OR #46 OR #47	180196
#31	'Middle East respiratory syndrome'/exp	3,392
	#37 OR #48 OR #49 OR #50 OR #51 OR #52 OR #53 OR #54(xuhao)	12895
#32	'ebola hemorrhagic fever'/exp	9,689
	#31 OR #55 OR #56 OR #57 OR #58 OR #59 OR #60 OR #61 OR #62 OR #63	10092
#33	'Marburg hemorrhagic fever'/exp	657
	#32 OR #64 OR #65 OR #66 OR #67 OR #68	646
#34	'Lassa fever'/exp	1494
	#32 OR #64 OR #65 OR #66 OR #67 OR #68	1178
#35	'Nipah virus infection'/exp	569
	#34 OR #75 OR #76	684
#36	'Zika fever'/exp	10,486
	#35 OR #77 OR #78 OR #79 OR #80 OR #81 OR #82 OR #83 OR #84 OR #85 OR #86 OR #87	10029
#37	'coronavirus disease 2019'/exp	448,070

	#36 OR #88 OR #89 OR #90 OR #91 OR #92 OR #93 OR #94 OR #95 OR #96 OR #97 OR #98 OR #99 OR #100 OR #101 OR #102 OR #103 OR #104 OR #105 OR #106 OR #107 OR #108 OR #109 OR #110 OR #111 OR #112 OR #113 OR #114 OR #115	445727
#38	'Communicable Disease, Emerging':ab,ti	1
#39	'Disease*, Emerging Communicable':ab,ti	0
#40	'Emerging Communicable Disease*':ab,ti	25
#41	'Infectious Diseases, Emerging':ab,ti	29
#42	'Disease*, Emerging Infectious':ab,ti	10
#43	'Emerging Infectious Diseases':ab,ti	3,731
#44	'Infectious Disease, Emerging':ab,ti	7
#45	'Respiratory Syndrome, Severe Acute':ab,ti	17
#46	'SARS':ab,ti	171,432
#47	'Respiratory Syndrome, Acute, Severe':ab,ti	0
#48	'MERS Virus*':ab,ti	59
#49	'Middle East respiratory syndrome-related coronavirus':ab,ti	61
#50	'Virus, MERS':ab,ti	90
#51	'Middle East respiratory syndrome related coronavirus':ab,ti	61
#52	'MERS-CoV':ab,ti	4,222
#53	'Merbecovirus*':ab,ti	55
#54	'MERS':ab,ti	9,480
#55	'Ebola Infection':ab,ti	191
#56	'Infection, Ebola':ab,ti	14
#57	'Ebola Virus Disease':ab,ti	3,138
#58	'Ebola Virus Infection':ab,ti	567
#59	'Infection, Ebola Virus':ab,ti	7
#60	'Virus Infection, Ebola':ab,ti	3
#61	'Ebolavirus Infection*':ab,ti	91
#62	'Infection*, Ebolavirus':ab,ti	0
#63	'Hemorrhagic Fever, Ebola':ab,ti	20
#64	'Marburg Virus Disease':ab,ti	213
#65	'Fever, Marburg Hemorrhagic':ab,ti	2
#66	'Hemorrhagic Fever, Marburg':ab,ti	9
#67	'Marburg Disease':ab,ti	58
#68	'Disease, Marburg':ab,ti	18
#69	'Fever, Lassa':ab,ti	28
#70	'Lassa Fevers':ab,ti	4
#71	'Lassa Virus Infection':ab,ti	78
#72	'Infection, Lassa Virus':ab,ti	0
#73	'Lassa Virus Infections':ab,ti	17
#74	'Virus Infection, Lassa':ab,ti	2
#75	'Nipah Viruses':ab,ti	98
#76	'Virus*, Nipah':ab,ti	85

#77	'Infection, Zika Virus':ab,ti	14
#78	'Virus Infection, Zika':ab,ti	6
#79	'ZikV Infection':ab,ti	3,167
#80	'Infection, ZikV':ab,ti	95
#81	'Fever, Zika':ab,ti	132
#82	'Zika Virus Disease':ab,ti	293
#83	'Disease, Zika Virus':ab,ti	9
#84	'Virus Disease, Zika':ab,ti	5
#85	'Zika Virus Infection':ab,ti	2,390
#86	'Congenital Zika Syndrome':ab,ti	843
#87	'Congenital Zika Virus Infection':ab,ti	182
#88	'2019-nCoV Infection*':ab,ti	229
#89	'Infection, SARS-CoV-2':ab,ti	415
#90	'Infection, 2019-nCoV':ab,ti	7
#91	'SARS-CoV-2 Infection*':ab,ti	46,656
#92	'SARS CoV 2 Infection':ab,ti	43,761
#93	'2019 Novel Coronavirus Disease':ab,ti	412
#94	'2019 Novel Coronavirus Infection':ab,ti	87
#95	'COVID-19 Virus Infection*':ab,ti	107
#96	'Infection, COVID-19 Virus':ab,ti	5
#97	'Virus Infection, COVID-19':ab,ti	40
#98	'COVID19':ab,ti	437,729
#99	'COVID-19':ab,ti	443,260
#100	'Disease 2019, Coronavirus':ab,ti	10
#101	'Coronavirus Disease-19':ab,ti	3,753
#102	'Coronavirus Disease 19':ab,ti	3,737
#103	'COVID-19 Virus Disease':ab,ti	9
#104	'Severe Acute Respiratory Syndrome Coronavirus 2 Infection ':ab,ti	1,464
#105	'COVID 19 Virus Disease ':ab,ti	9
#106	'COVID-19 Virus Diseases':ab,ti	0
#107	'Disease, COVID-19 Virus':ab,ti	7
#108	'Virus Disease, COVID-19':ab,ti	335
#109	'SARS Coronavirus 2 Infection':ab,ti	17
#110	'2019 nCoV Disease':ab,ti	21
#111	'2019-nCoV Disease*':ab,ti	23
#112	'Disease, 2019-nCoV':ab,ti	17
#113	'COVID-19 Pandemic*':ab,ti	173,223
#114	'Pandemic, COVID-19':ab,ti	1,921
#115	'COVID 19 Pandemic':ab,ti	172,535
#116	#29 OR #30 OR #31 OR #32 OR #33 OR #34 OR #35 OR #36 OR #37 OR #38 OR #39 OR #40 OR #41 OR #42 OR #43 OR #44 OR #45 OR #46 OR #47 OR #48 OR #49 OR #50 OR #51 OR #52 OR #53 OR #54 OR #55 OR #56 OR #57 OR #58 OR #59 OR #60 OR #61 OR #62 OR #63 OR #64 OR #65 OR #66 OR	595,657

	#67 OR #68 OR #69 OR #70 OR #71 OR #72 OR #73 OR #74 OR #75 OR #76 OR #77 OR #78 OR #79 OR #80 OR #81 OR #82 OR #83 OR #84 OR #85 OR #86 OR #87 OR #88 OR #89 OR #90 OR #91 OR #92 OR #93 OR #94 OR #95 OR #96 OR #97 OR #98 OR #99 OR #100 OR #101 OR #102 OR #103 OR #104 OR #105 OR #106 OR #107 OR #108 OR #109 OR #110 OR #111 OR #112 OR #113 OR #114 OR #115	
#117	#7 AND #28 AND #116	131
**CINAHL**		
S1	(MH "Nurse Administrators") OR (TI ("Administrator, Nurse" OR "Administrators, Nurse" OR "Nurse Administrator" OR "Nurse Managers" OR "Manager, Nurse" OR "Managers, Nurse" OR "Nurse Manager" OR "Nurse Executives" OR "Executive, Nurse" OR "Executives, Nurse" OR "Nurse Executive")	7534
S2	(MH "Qualitative Studies+" OR "Focus Groups" OR "Grounded Theory" OR "Anthropology, Cultural") OR (TI ("Qualitative Research" OR "Research, Qualitative" OR "qualitative method" OR "qualitative stud*" OR "focus group" OR "Group, Focus" OR "Groups, Focus" OR "Grounded Theory" OR "Theory, Grounded" OR Ethnography OR Enthnograph* OR "Cultural Anthropology" OR "Material Culture" OR "Culture, Material" OR "Material Cultures" OR Ethnography OR Ethnographies OR Interview OR Phenomenol* OR discourse OR "Content analysis"))	221151
S3	(MH "Severe acute respiratory syndrome" OR "Middle East Respiratory Syndrome" OR "Hemorrhagic Fever, Ebola" OR "Zika Virus Infection" OR "COVID-19" OR (TI ("Lassa Fever" OR "Communicable Diseases, Emerging" OR "Marburg Virus Disease" OR "Nipah Virus" OR "Communicable Disease, Emerging" OR "Disease*, Emerging Communicable" OR "Emerging Communicable Disease*" OR "Infectious Diseases, Emerging" OR "Disease*, Emerging Infectious" OR "Emerging Infectious Disease*" OR "Infectious Disease, Emerging" OR "Respiratory Syndrome, Severe Acute" OR "SARS" OR "Respiratory Syndrome, Acute, Severe" OR "MERS Virus*" OR "Virus, MERS" OR "Middle East respiratory syndrome-related coronavirus" OR "Middle East respiratory syndrome related coronavirus" OR "MERS-CoV" OR "Merbecovirus*" OR "Ebola Infection" OR "Infection, Ebola" OR "Ebola Virus Disease" OR "Ebola Virus Infection" OR "Infection, Ebola Virus" OR "Virus Infection, Ebola" OR "Ebolavirus Infection*" OR "Infection*, Ebolavirus" OR "Ebola Hemorrhagic Fever" OR "Marburg Hemorrhagic Fever" OR "Fever, Marburg Hemorrhagic" OR "Hemorrhagic Fever, Marburg" OR "Marburg Disease" OR "Disease, Marburg" OR "Fever, Lassa" OR "Lassa Fevers" OR "Lassa Virus Infection" OR "Infection, Lassa Virus" OR "Lassa Virus Infections" OR "Virus Infection, Lassa" OR "Nipah Viruses" OR "Virus*, Nipah" OR "Infection, Zika Virus" OR "Virus Infection, Zika" OR "ZikV Infection" OR "Infection, ZikV" OR "Fever, Zika" OR "Zika Virus Disease" OR "Disease, Zika Virus" OR "Virus Disease, Zika" OR "Zika Fever" OR "Congenital Zika Syndrome" OR "Congenital Zika Virus Infection" OR	137585

	"2019-nCoV Infection*" OR "Infection, 2019-nCoV" OR "SARS-CoV-2 Infection*" OR "Infection, SARS-CoV-2" OR "SARS CoV 2 Infection" OR "2019 Novel Coronavirus Disease" OR "2019 Novel Coronavirus Infection" OR "COVID-19 Virus Infection*" OR "Infection, COVID-19 Virus" OR "Virus Infection, COVID-19" OR "COVID19" OR "Coronavirus Disease 2019" OR "Disease 2019, Coronavirus" OR "Coronavirus Disease-19" OR "Coronavirus Disease 19" OR "Severe Acute Respiratory Syndrome Coronavirus 2 Infection" OR "COVID-19 Virus Disease" OR "COVID 19 Virus Disease" OR "COVID-19 Virus Diseases" OR "Disease, COVID-19 Virus" OR "Virus Disease, COVID-19" OR "SARS Coronavirus 2 Infection" OR "2019-nCoV Disease*" OR "2019 nCoV Disease" OR "Disease, 2019-nCoV" OR "COVID-19 Pandemic*" OR "COVID 19 Pandemic" OR "Pandemic, COVID-19"	
S4	S1 AND S2 AND S3	35
**Web of science**		
#1	TS=(Nurse Administrators) OR (Administrator, Nurse) OR (Administrators, Nurse) OR (Nurse Administrator) OR (Nurse Managers) OR (Manager, Nurse) OR (Managers, Nurse) OR (Nurse Manager) OR (Nurse Executives) OR (Executive, Nurse) OR (Executives, Nurse) OR (Nurse Executive)	47,130
#2	TS=(Qualitative Research OR Research, Qualitative OR qualitative method OR qualitative stud* OR focus group OR Focus Group OR Group, Focus OR Groups, Focus OR Grounded Theory OR Theory, Grounded OR Ethnography OR Enthnograph* OR Cultural Anthropology OR Material Culture OR Culture, Material OR Material Cultures OR Ethnography OR Ethnographies OR Interview OR Phenomenol* OR discourse OR Content analysis)	4,087,005
#3	TS=(Communicable Diseases, Emerging) OR (Severe acute respiratory syndrome) OR (Middle East Respiratory Syndrome Coronavirus) OR (Hemorrhagic Fever, Ebola) OR (Marburg Virus Disease) OR (Lassa Fever) OR (Nipah Virus) OR (Zika Virus Infection) OR (COVID-19) OR (Communicable Disease, Emerging) OR (Disease*, Emerging Communicable) OR (Emerging Communicable Disease*) OR (Infectious Diseases, Emerging) OR (Disease*, Emerging Infectious) OR (Emerging Infectious Disease*) OR (Infectious Disease, Emerging)) OR (Respiratory Syndrome, Severe Acute) OR (SARS) OR (Respiratory Syndrome, Acute, Severe) OR (MERS Virus*) OR (Virus, MERS) OR (Middle East respiratory syndrome-related coronavirus) OR (Middle East respiratory syndrome related coronavirus) OR (MERS-CoV) OR (Merbecovirus*) OR (Ebola Infection) OR (Infection, Ebola) OR (Ebola Virus Disease) OR (Ebola Virus Infection) OR (Infection, Ebola Virus) OR (Virus Infection, Ebola) OR (Ebolavirus Infection*) OR (Infection*, Ebolavirus) OR (Ebola Hemorrhagic Fever) OR (Marburg Hemorrhagic Fever) OR (Fever, Marburg Hemorrhagic) OR (Hemorrhagic Fever, Marburg) OR (Marburg Disease) OR (Disease, Marburg) OR (Fever, Lassa) OR (Lassa Fevers) OR (Lassa Virus Infection) OR (Infection, Lassa Virus) OR (Lassa Virus Infections) OR (Virus Infection, Lassa) OR (Nipah Viruses) OR (Virus*,	771,415

	Nipah) OR (Infection, Zika Virus) OR (Virus Infection, Zika) OR (ZikV Infection) OR (Infection, ZikV) OR (Fever, Zika) OR (Zika Virus Disease) OR (Disease, Zika Virus) OR (Virus Disease, Zika) OR (Zika Fever) OR (Congenital Zika Syndrome) OR (Congenital Zika Virus Infection) OR (2019-nCoV Infection*) OR (Infection, 2019-nCoV) OR (SARS-CoV-2 Infection*) OR (Infection, SARS-CoV-2) OR (SARS CoV 2 Infection) OR (2019 Novel Coronavirus Disease) OR (2019 Novel Coronavirus Infection) OR (COVID-19 Virus Infection*) OR (Infection, COVID-19 Virus) OR (Virus Infection, COVID-19) OR (COVID19) OR (Coronavirus Disease 2019) OR (Disease 2019, Coronavirus) OR (Coronavirus Disease-19) OR (Coronavirus Disease 19) OR (Severe Acute Respiratory Syndrome Coronavirus 2 Infection) OR (COVID-19 Virus Disease) OR (COVID 19 Virus Disease) OR (COVID-19 Virus Diseases) OR (Disease, COVID-19 Virus) OR (Virus Disease, COVID-19) OR (SARS Coronavirus 2 Infection) OR (2019-nCoV Disease*) OR (2019 nCoV Disease) OR (Disease, 2019-nCoV) OR (COVID-19 Pandemic*) OR (COVID 19 Pandemic) OR (Pandemic, COVID-19)	
#4	#1 AND #2 AND #3	698
**Cochrane**		
#1	MeSH descriptor: [Nurse Administrators] explode all trees	44
#2	(dysphagia OR Deglutition Disorder OR Disorders, Deglutition OR Swallowing Disorders OR Swallowing Disorder OR Dysphagia OR Oropharyngeal Dysphagia OR Dysphagia, Oropharyngeal OR Esophageal Dysphagia OR Dysphagia, Esophageal):ti,ab,kw<br>(Administrator, Nurse) OR (Administrators, Nurse) OR (Nurse Administrator) OR (Nurse Managers) OR (Manager, Nurse) OR (Managers, Nurse) OR (Nurse Manager) OR (Nurse Executives) OR (Executive, Nurse) OR (Executives, Nurse) OR (Nurse Executive):ti,ab,kw	22107
#3	#1 OR #2	22107
#4	MeSH descriptor: [Qualitative Research] explode all trees	2532
#5	MeSH descriptor: [Focus group] explode all trees	1128
#6	MeSH descriptor: [Grounded Theory] explode all trees	28
#7	MeSH descriptor: [Anthropology, Cultural] explode all trees	3826
#8	(Qualitative Research OR Research, Qualitative OR qualitative method OR qualitative stud* OR focus group OR Focus Groups OR Group, Focus OR Groups, Focus OR Grounded Theory OR Theory, Grounded OR Ethnography OR Enthnograph* OR Cultural Anthropology OR Material Culture OR Culture, Material OR Material Cultures OR Ethnography OR Ethnographies OR Interview OR Phenomenol* OR discourse OR Content analysis):ti,ab,kw	122430
#9	#4 OR #5 OR #6 OR #7 OR #8	122430
#10	MeSH descriptor: [Communicable Diseases, Emerging] explode all trees	18
#11	MeSH descriptor: [Severe acute respiratory syndrome] explode all trees	267
#12	MeSH descriptor: [Middle East Respiratory Syndrome Coronavirus] explode all trees	11
#13	MeSH descriptor: [Hemorrhagic Fever, Ebola] explode all trees	175

#14	MeSH descriptor: [Marburg Virus Disease] explode all trees	12
#15	MeSH descriptor: [Lassa Fever] explode all trees	12
#16	MeSH descriptor: [Nipah Virus] explode all trees	2
#17	MeSH descriptor: [Zika Virus Infection] explode all trees	64
#18	MeSH descriptor: [COVID-19] explode all trees	8390
#19	(Communicable Disease, Emerging OR Disease*, Emerging Communicable OR Emerging Communicable Disease* OR Infectious Diseases, Emerging OR Disease*, Emerging Infectious OR Emerging Infectious Disease* OR Infectious Disease, Emerging):ti,ab,kw	1861
#20	(Respiratory Syndrome, Severe Acute OR SARS OR Respiratory Syndrome, Acute, Severe):ti,ab,kw	11685
#21	(MERS Virus* OR Virus, MERS OR Middle East respiratory syndrome-related coronavirus OR Middle East respiratory syndrome related coronavirus OR MERS-CoV OR Merbecovirus*):ti,ab,kw	153
#22	(Ebola Infection OR Infection, Ebola OR Ebola Virus Disease OR Ebola Virus Infection OR Infection, Ebola Virus OR Virus Infection, Ebola OR Ebolavirus Infection* OR Infection*, Ebolavirus OR Ebola Hemorrhagic Fever):ti,ab,kw	330
#23	(Marburg Hemorrhagic Fever OR Fever, Marburg Hemorrhagic OR Hemorrhagic Fever, Marburg OR Marburg Disease OR Disease, Marburg):ti,ab,kw	57
#24	(Fever, Lassa OR Lassa Fevers OR Lassa Virus Infection OR Infection, Lassa Virus OR Lassa Virus Infections OR Virus Infection, Lassa):ti,ab,kw	29
#25	(Nipah Viruses OR Virus*, Nipah):ti,ab,kw	10
#26	(Infection, Zika Virus OR Virus Infection, Zika OR ZikV Infection OR Infection, ZikV OR Fever, Zika OR Zika Virus Disease OR Disease, Zika Virus OR Virus Disease, Zika OR Zika Fever OR Congenital Zika Syndrome OR Congenital Zika Virus Infection):ti,ab,kw	145
#27	(SARS-CoV-2 Infection* OR Infection, SARS-CoV-2 OR SARS CoV 2 Infection OR 2019 Novel Coronavirus Disease OR 2019 Novel Coronavirus Infection OR COVID-19 Virus Infection* OR Infection, COVID-19 Virus OR Virus Infection, COVID-19 OR COVID19 OR Coronavirus Disease 2019 OR Disease 2019, Coronavirus OR Coronavirus Disease-19 OR Coronavirus Disease 19 OR Severe Acute Respiratory Syndrome Coronavirus 2 Infection OR COVID-19 Virus Disease OR COVID 19 Virus Disease OR COVID-19 Virus Diseases OR Disease, COVID-19 Virus OR Virus Disease, COVID-19 OR SARS Coronavirus 2 Infection OR 2019 nCoV Disease OR COVID-19 Pandemic* OR COVID 19 Pandemic OR Pandemic, COVID-19):ti,ab,kw	16876
#28	#10 OR #11 OR #12 OR #13 OR #14 OR #15 OR #16 OR #17 OR #18 OR #19 OR #20 OR #21 OR #22 OR #23 OR #24 OR #25 OR #26 OR #27	24623
#29	#3 AND #9 AND #28	194
CNKI		
	SU=('emerging infectious diseases'+'emerging infectious disease outbreaks'+'SARS'+'severe acute respiratory syndrome'+'Middle East respiratory syndrome'+'Ebola'+'Marburg virus disease'+'Lassa fever'+'Lassa	10

	hemorrhagic fever'+'Nipah virus'+'Zika virus'+'COVID-19'+'novel coronavirus pneumonia'+'coronavirus disease 2019') AND SU=('qualitative research'+'grounded theory'+'ethnography') AND SU=('head nurses'+'nursing managers')	
	VIP	
	((Title or Keywords=(emerging infectious diseases OR emerging infectious disease outbreaks OR SARS OR atypical pneumonia OR severe acute respiratory syndrome OR Middle East respiratory syndrome OR Ebola OR Marburg virus disease OR Lassa fever OR Lassa hemorrhagic fever OR Nipah virus OR Zika virus OR COVID-19 OR novel coronavirus pneumonia OR coronavirus disease 2019)) AND Title or Keywords=(head nurses OR nursing managers)) AND Title or Keywords=(qualitative research OR grounded theory OR ethnography)	2
	Wanfang	
	((Title or Keywords=(emerging infectious diseases OR emerging infectious disease outbreaks OR SARS OR atypical pneumonia OR severe acute respiratory syndrome OR Middle East respiratory syndrome OR Ebola OR Marburg virus disease OR Lassa fever OR Lassa hemorrhagic fever OR Nipah virus OR Zika virus OR COVID-19 OR novel coronavirus pneumonia OR coronavirus disease 2019)) AND Title or Keywords=(head nurses OR nursing managers)) AND Title or Keywords=(qualitative research OR grounded theory OR ethnography)	2
	ProQuest	
S1	ti,ab((Nurse Administrators) OR (Administrator, Nurse) OR (Administrators, Nurse) OR (Nurse Administrator) OR (Nurse Managers) OR (Manager, Nurse) OR (Managers, Nurse) OR (Nurse Manager) OR (Nurse Executives) OR (Executive, Nurse) OR (Executives, Nurse) OR (Nurse Executive))	16,137
#2	ti,ab(Qualitative Research OR Research, Qualitative OR qualitative method OR qualitative stud* OR focus group OR Focus Group OR Group, Focus OR Groups, Focus OR Grounded Theory OR Theory, Grounded OR Ethnography OR Enthnograph* OR Cultural Anthropology OR Material Culture OR Culture, Material OR Material Cultures OR Ethnography OR Ethnographies OR Interview OR Phenomenol* OR discourse OR Content analysis)	667,870
#3	ti,ab(Communicable Diseases, Emerging OR Severe acute respiratory syndrome OR Middle East Respiratory Syndrome Coronavirus OR Hemorrhagic Fever, Ebola OR Marburg Virus Disease OR lass Fever OR nipa Virus OR Zika Virus Infection OR COVID-19 OR Communicable Disease, Emerging OR Disease*, Emerging Communicable OR Emerging Communicable Disease* OR Infectious Diseases, Emerging OR Disease*, Emerging Infectious OR Emerging Infectious Disease* OR Infectious Disease, Emerging OR Respiratory Syndrome, Severe Acute OR SARS OR Respiratory Syndrome, Acute, Severe OR MERS Virus* OR Virus, MERS OR Middle East respiratory syndrome-related coronavirus OR Middle East respiratory syndrome related coronavirus OR MERS-CoV OR Merbecovirus* OR Ebola Infection OR Infection, Ebola OR Ebola Virus Disease OR Ebola Virus Infection OR Infection, Ebola Virus OR Virus Infection, Ebola OR Ebolavirus Infection* OR Infection*, Ebolavirus OR Ebola Hemorrhagic	111,776

	Fever OR Marburg Hemorrhagic Fever OR Fever, Marburg Hemorrhagic OR Hemorrhagic Fever, Marburg OR Marburg Disease OR Fever, lass OR lass Fevers OR lass Virus Infection OR Infection, lass Virus OR lass Virus Infections OR Virus Infection, lass OR nipa Viruses OR Virus*, nipa OR Infection, Zika Virus OR Virus Infection, Zika OR ZikV Infection OR Infection, ZikV OR Fever, Zika OR Zika Virus Disease OR Disease, Zika Virus OR Virus Disease, Zika OR Zika Fever OR Congenital Zika Syndrome OR Congenital Zika Virus Infection OR 2019-nCoV Infection* OR Infection, 2019-nCoV OR SARS-CoV-2 Infection* OR Infection, SARS-CoV-2 OR SARS CoV 2 Infection OR 2019 Novel Coronavirus Disease OR 2019 Novel Coronavirus Infection OR COVID-19 Virus Infection* OR Infection, COVID-19 Virus OR Virus Infection, COVID-19 OR COVID19 OR Coronavirus Disease 2019 OR Disease 2019, Coronavirus OR Coronavirus Disease-19 OR Coronavirus Disease 19 OR Severe Acute Respiratory Syndrome Coronavirus 2 Infection OR COVID-19 Virus Disease OR COVID 19 Virus Disease OR COVID-19 Virus Diseases OR Disease, COVID-19 Virus OR Virus Disease, COVID-19 OR SARS Coronavirus 2 Infection OR 2019-nCoV Disease* OR 2019 nCoV Disease OR Disease, 2019-nCoV OR COVID-19 Pandemic* OR COVID 19 Pandemic OR Pandemic, COVID-19)	
#4	#1 AND #2 AND #3	44
	Scopus	
#1	(TITLE-ABS-KEY ("nursing manager*" OR "nurse administrator*" OR "nursing supervisor*" OR "clinical nurse leader*" OR "head nurse"))	27417
#2	(TITLE-ABS-KEY ("emerging infectious disease*" OR "novel infectious disease*" OR "outbreak*" OR "epidemic*" OR "pandemic*" OR "COVID-19" OR "SARS" OR "Ebola" OR "MERS" OR "Zika virus"))	1248548
#3	(TITLE-ABS-KEY ("nursing care experience*" OR "emergency care" OR "clinical experience*" OR "crisis intervention" OR "patient care" OR "emergency response"))	647864
#4	(TITLE-ABS-KEY ("training need*" OR "education need*" OR "competency development" OR "skill gap*" OR "professional development" OR "capacity building"))	127977
	#1 AND #2 AND #3 AND #4	4
	Chinese Biomedical Literature Database (SinoMed)	
#1	Nursing manager [Intelligent Search] OR Head nurse [Intelligent Search] OR Director of nursing department [Intelligent Search] OR Nursing supervisor [Intelligent Search] OR Nursing management personnel [Intelligent Search] OR "Nursing managers" [Subject Heading]	46012
#2	Emerging infectious diseases [Intelligent Search] OR Emerging communicable diseases [Intelligent Search] OR Infectious disease outbreak [Intelligent Search] OR Epidemic [Intelligent Search] OR Coronavirus disease 2019 (COVID-19) [Intelligent Search] OR COVID-19 [Intelligent Search] OR Severe Acute Respiratory Syndrome (SARS) [Intelligent Search] OR Ebola [Intelligent Search]	563494

#3	Rescue care [Intelligent Search] OR Nursing care [Intelligent Search] OR Response [Intelligent Search]) AND (Experience [Intelligent Search] OR Lessons learned [Intelligent Search] OR Feelings [Intelligent Search] OR Challenges [Intelligent Search]	3141645
#4	Training needs [Intelligent Search] OR Educational needs [Intelligent Search] OR Capacity building [Intelligent Search] OR Skills training [Intelligent Search] OR Continuing education [Intelligent Search]	107951
#5	#1 AND #2 AND #3 AND #4	16
